# Supplementary material for: Intestinal stem cell aging at single‐cell resolution: Transcriptional perturbations alter cell developmental trajectory reversed by gerotherapeutics
Source: Aging Cell. 2023 Mar 2;22(5):e13802. doi: 10.1111/acel.13802 (PMC10186593; doi:10.1111/acel.13802)
Supplement: Supplementary file 1 — Figure S1. Figure S2. Figure S3. Figure S4. [file ACEL-22-e13802-s001.pdf]

Supplementary figures

Figure S1

a FACS gating stretagy for total epithelial cells (Epcam+, CD45neg)

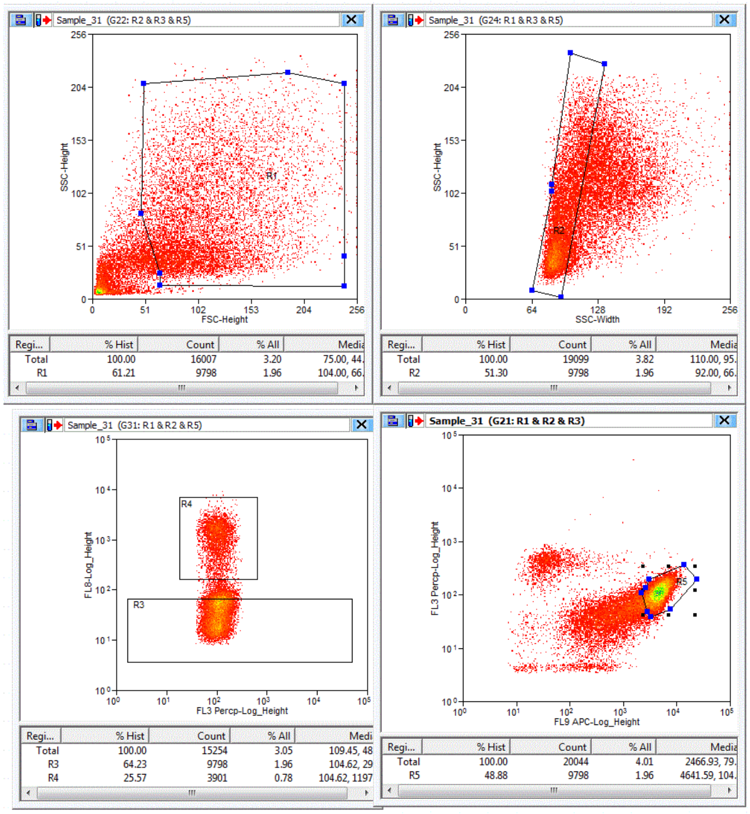

b Expression of cell type and lineage markers in cell clusters

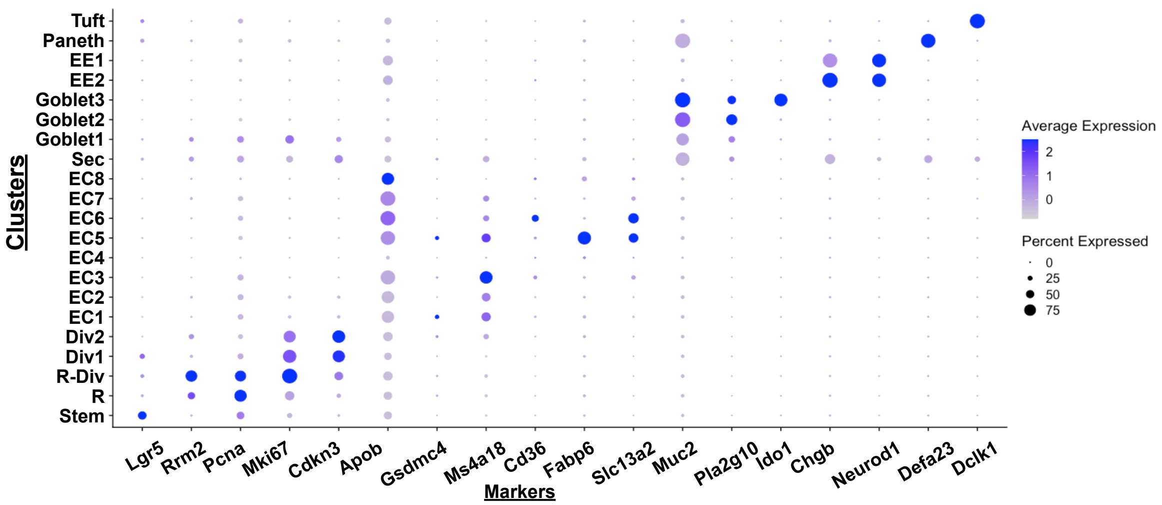

c Fraction of total cells in each cluster

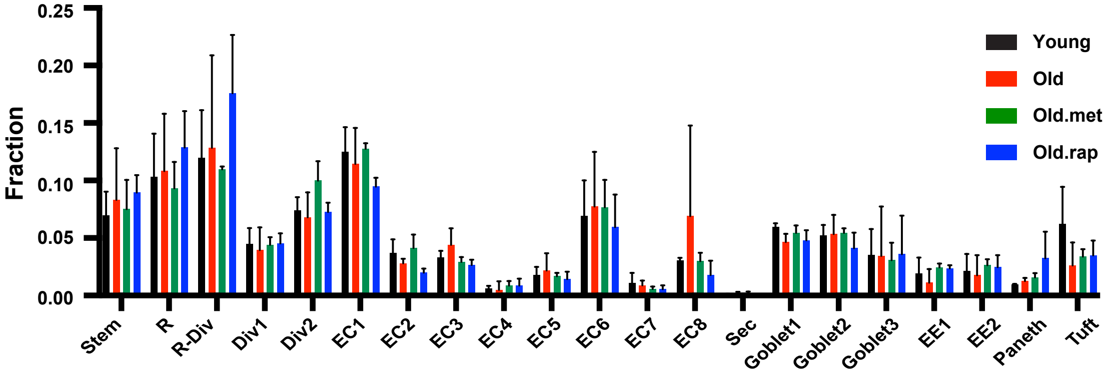

Figure S2

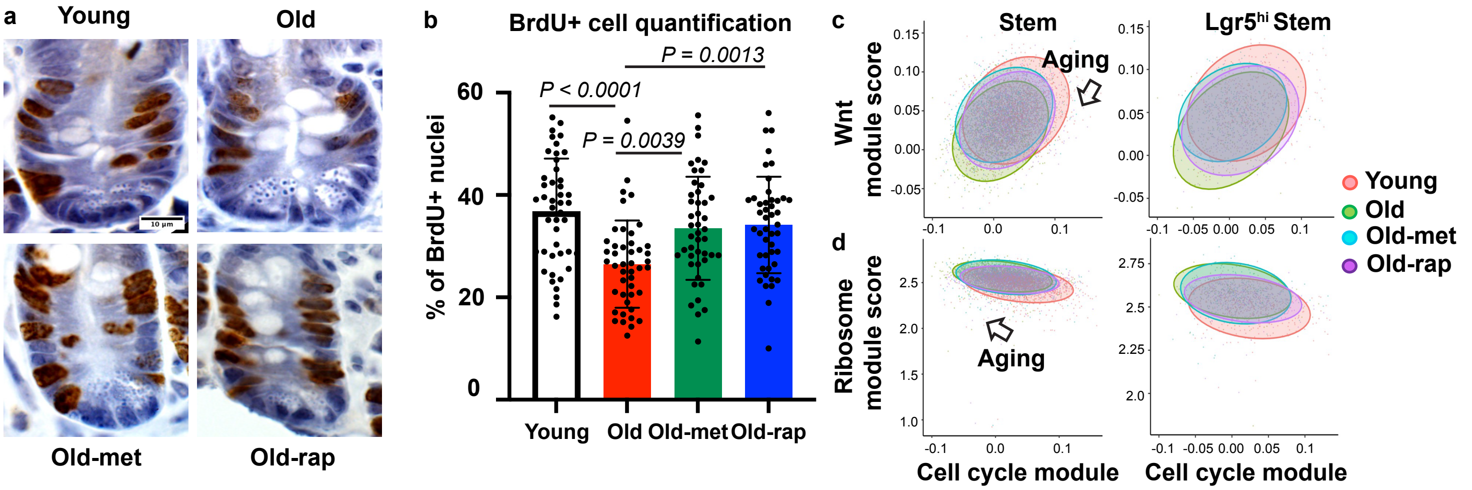
a
Young
Old
Old-met
Old-rap
10 μm
b
BrdU+ cell quantification
% of BrdU+ nuclei
P < 0.0001
P = 0.0039
P = 0.0013
0
20
40
60
Young
Old
Old-met
Old-rap
c
Stem
Lgr5hi Stem
Wnt module score
Aging
d
Ribosome module score
Aging
Cell cycle module
Cell cycle module
Young
Old
Old-met
Old-rap

Figure S3

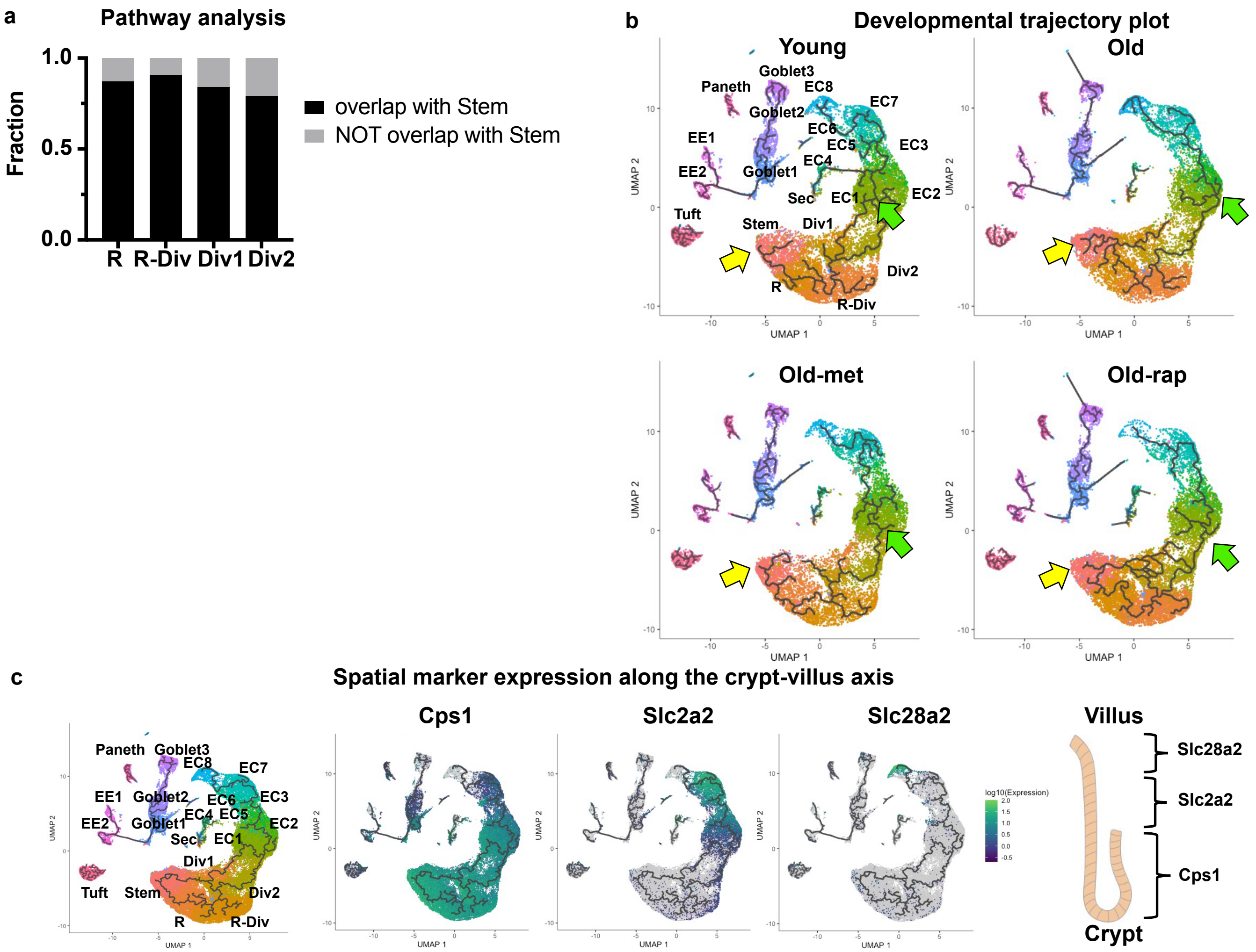

a
Pathway analysis
Fraction
1.0
0.5
0.0
R
R-Div
Div1
Div2
overlap with Stem
NOT overlap with Stem
b
Developmental trajectory plot
Young
Old
Old-met
Old-rap
Paneth
Goblet3
Goblet2
Goblet1
EE1
EE2
Tuft
Stem
Sec
Div1
Div2
R
R-Div
EC1
EC2
EC3
EC4
EC5
EC6
EC7
EC8
UMAP 1
UMAP 2
c
Spatial marker expression along the crypt-villus axis
Cps1
Slc2a2
Slc28a2
log10(Expression)
Villus
Crypt

Figure S4

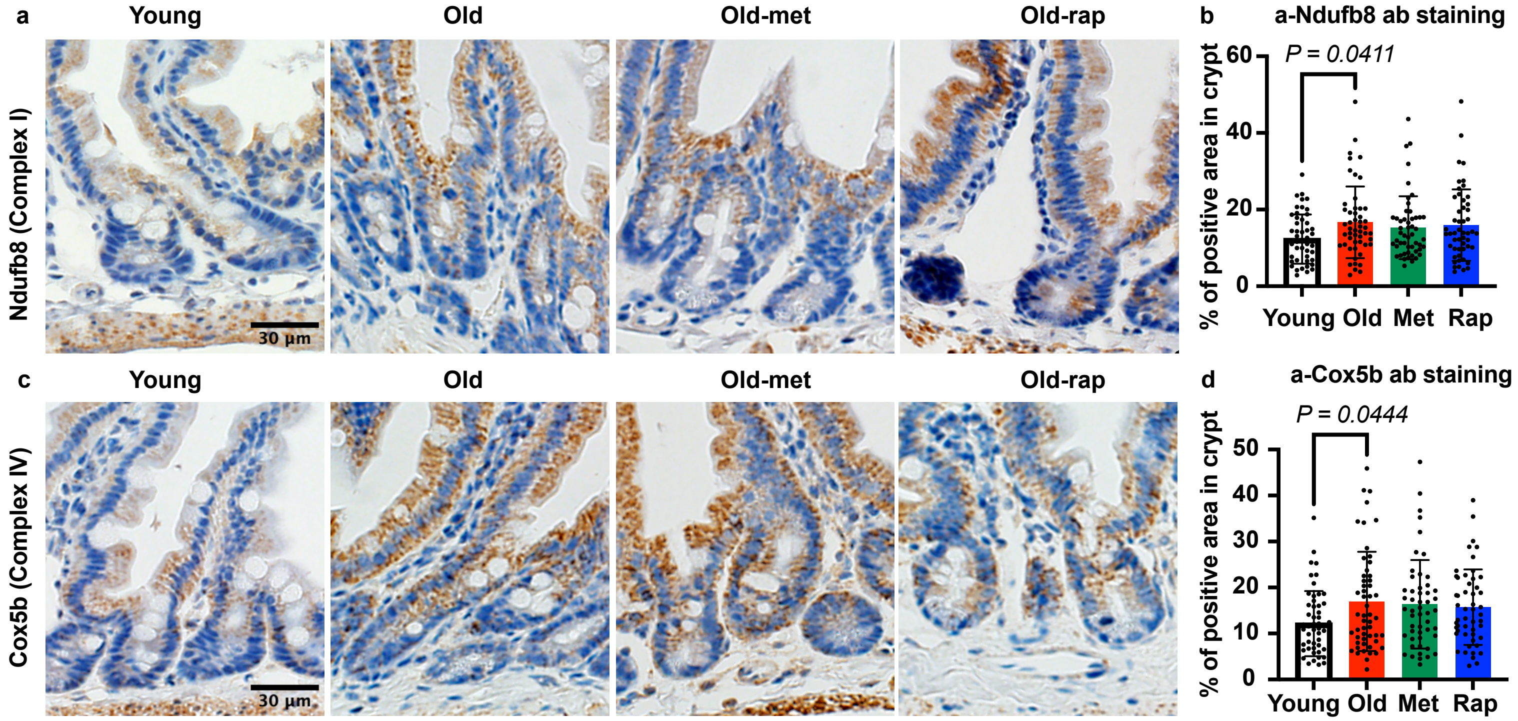
a
Young
Old
Old-met
Old-rap
Ndufb8 (Complex I)
30 µm
b
a-Ndufb8 ab staining
P = 0.0411
% of positive area in crypt
60
40
20
0
Young
Old
Met
Rap
c
Young
Old
Old-met
Old-rap
Cox5b (Complex IV)
30 µm
d
a-Cox5b ab staining
P = 0.0444
% of positive area in crypt
50
40
30
20
10
0
Young
Old
Met
Rap
