# Supplementary material for: Intestinal stem cell aging at single‐cell resolution: Transcriptional perturbations alter cell developmental trajectory reversed by gerotherapeutics
Source: Aging Cell. 2023 Mar 2;22(5):e13802. doi: 10.1111/acel.13802 (PMC10186593; doi:10.1111/acel.13802)
Supplement: Supplementary file 2 — Table S1. Table S2. [file ACEL-22-e13802-s002.docx]

**Table S1. Number of differentially expressed genes**

| **celltype** | **differential genes**  **O vs Y** | **differential genes**  **O-met vs O** | **differential genes**  **O-rap vs O** |
| --- | --- | --- | --- |
| **Stem** | 68 | 48 | 31 |
| **R** | 62 | 40 | 24 |
| **R_Div** | 80 | 39 | 29 |
| **Div1** | 69 | 46 | 21 |
| **Div2** | 58 | 37 | 24 |
| **EC1** | 82 | 28 | 33 |
| **EC2** | 76 | 40 | 39 |
| **EC3** | 159 | 79 | 38 |
| **EC4** | 8 | 5 | 2 |
| **EC5** | 30 | 63 | 126 |
| **EC6** | 94 | 140 | 124 |
| **EC7** | 78 | 28 | 39 |
| **EC8** | 564 | 725 | 838 |
| **Sec** | 57 | 24 | 24 |
| **Goblet1** | 31 | 18 | 41 |
| **Goblet2** | 40 | 52 | 110 |
| **Goblet3** | 6 | 2 | 37 |
| **EE1** | 9 | 3 | 9 |
| **EE2** | 31 | 9 | 24 |
| **Paneth** | 76 | 6 | 52 |
| **Tuft** | 2 | 19 | 15 |

**Table S2. Multiple comparison for Figure 3b-i**

|  |  | **Significance** | | | |
| --- | --- | --- | --- | --- | --- |
|  |  | **Wnt** | **Cell cycle** | **Ribosome** | **OXPHOS** |
| **main** | **Y vs O** | ******** | ******** | ***** | ****** |
|  | **Y vs O-met** | **ns** | ****** | **ns** | **ns** |
|  | **Y vs O-rap** | ******* | ****** | **ns** | ****** |
|  | **O vs O-met** | ******* | ****** | **ns** | ***** |
|  | **O vs O-rap** | **ns** | ****** | **ns** | **ns** |
|  | **O-met vs O-rap** | ****** | **ns** | **ns** | ***** |
| **side** | **Y vs O** | **ns** | **ns** | **ns** | **ns** |
|  | **Y vs O-met** | **ns** | ***** | **ns** | **ns** |
|  | **Y vs O-rap** | **ns** | **ns** | **ns** | **ns** |
|  | **O vs O-met** | **ns** | **ns** | **ns** | **ns** |
|  | **O vs O-rap** | **ns** | **ns** | **ns** | **ns** |
|  | **O-met vs O-rap** | **ns** | **ns** | **ns** | ****** |

| ***P < 0.0001*** | ******** |
| --- | --- |
| ***P < 0.001*** | ******* |
| ***P < 0.01*** | ****** |
| ***P < 0.05*** | ***** |
